# Transitions from hospital to community care: the role of patient–provider language concordance

**DOI:** 10.1186/2045-4015-3-24

**Published:** 2014-07-22

**Authors:** Nosaiba Rayan, Hanna Admi, Efrat Shadmi

**Affiliations:** 1 School of Public Health, Faculty of Social Welfare and Health Sciences, Haifa University, Mount Carmel 31905, Israel; 2 Rambam Medical Campus: Nursing Division, Haifa 31096, Israel; 3 The Cheryl Spencer Department of Nursing, Faculty of Social Welfare and Health Sciences, Haifa University, Mount Carmel 31905, Israel

**Keywords:** Transitional care, Language concordance, Cultural differences, Quality of care, Minority patients

## Abstract

**Background:**

Cultural and language discordance between patients and providers constitutes a significant challenge to provision of quality healthcare. This study aims to evaluate minority patients’ discharge from hospital to community care, specifically examining the relationship between patient–provider language concordance and the quality of transitional care.

**Methods:**

This was a multi-method prospective study of care transitions of 92 patients: native Hebrew, Russian or Arabic speakers, with a pre-discharge questionnaire and structured observations examining discharge preparation from a large Israeli teaching hospital. Two weeks post-discharge patients were surveyed by phone, on the transition from hospital to community care (the Care Transition Measure (CTM-15, 0–100 scale)) and on the primary-care post-discharge visit.

**Results:**

Overall, ratings on the CTM indicated fair quality of the transition process (scores of 51.8 to 58.8). Patient–provider language concordance was present in 49% of minority patients’ discharge briefings. Language concordance was associated with higher CTM scores among minority groups (64.1 in language-concordant versus 49.8 in non-language-concordant discharges, *P* <0.001). Other aspects significantly associated with CTM scores: extent of discharge explanations (*P* <0.05), quality of discharge briefing (*P* <0.001), and post-discharge explanations by the primary care physician (*P* <0.01).

**Conclusion:**

Language-concordant care, coupled with extensive discharge briefings and post-discharge explanations for ongoing care, are important contributors to the quality of care transitions of ethnic minority patients.

## Background

Transition from the hospital to the community is a particularly vulnerable stage of the care trajectory due to the likelihood of breakdowns in communication and continuity of care, including omissions, duplications, or mismatched treatment recommendations [1]. Health care systems worldwide implement interventions to meet patients’ transitional care needs to improve post-hospitalization outcomes [2-6]. Tailoring care to meet patients’ needs in the transition is an important part of these interventions, as patients need to fully comprehend and perform their post-discharge care instructions.

Patients of ethnically diverse backgrounds are most vulnerable to breakdowns in the transition process due to language and cultural barriers that hinder their understanding of instructions for ongoing care [7]. Language and cultural issues that can affect the interaction between patients and providers, the ability to understand and apply the instructions for ongoing care include language concordance between patients and providers, religious beliefs, social relationships, communication styles, perception of the care and the health system [8,9], providers’ level of cultural competence [10] and patients’ health literacy [11,12].

When patients receive language-concordant care congruent with their culture and norms, they experience less language and cultural barriers. Language concordance between providers and their patients has proved an important contributor to the quality of care [13-17], associated with positive health outcomes for minorities [18]. A wide body of literature shows that language differences between patients and providers put patients at risk for a variety of poor outcomes, including lack of diabetes control [19], poor medication compliance [20] and dissatisfaction with emergency room visits [21]. However, to date studies have not tested the relation of patient–provider language concordance in care transitions with the quality of the transition process.

Israel’s diverse society and multi-ethnic population, with its national healthcare service and comprehensive primary care [22] offers a valuable setting for addressing this gap. Our study was conducted with Israeli *heterogeneous minority groups* among oncology patients: non-native Hebrew speakers of Arabic- or Russian-speaking backgrounds, and native Hebrew speakers.

All Israeli residents are covered by mandatory health insurance, financed mainly by a progressive health tax and provided by one of four health funds, operating as insurers and providers [23]. Previous Israeli studies have shown that disadvantaged groups face more barriers to specialty care than the rest of the population [24,25]. Israel’s historical development produced a country with three major population groups: Jews born in Israel or residing in Israel most of their lives (henceforward Hebrew speakers); immigrants from countries of the former Soviet Union (FSU); and Arabs. The majority of Hebrew speakers differ from the FSU immigrants (13.3% of the population in 2009) in background, culture and language. The Russian-speaking immigrants have unique characteristics, perceptions, patterns of attitudes and beliefs related to their country of origin [26]. The immigration process is a significant event with major changes in social environment, employment, income, living conditions and other factors, which affect many aspects of a person’s social and health conditions. In addition, FSU immigrants differ from the majority population in their health care use and their perceptions of care and the health care system [27,28].

Arabs living in Israel comprised 19.8% of the population in 2009. Arabs and Jews differ in ethnicity, religion, culture and language [29]. Arabic speakers have religious, ethnic, genetic and social characteristics, and a different lifestyle from Hebrew speakers. These characteristics are reflected in patterns of morbidity and mortality, use of health services and unique health perceptions [30]. With the exception of primary care, which is usually provided to Arab-speakers by Arabic speaking PCPs, minority patients in Israel often experience difficulty in communication with providers due to low Hebrew language proficiency [31,32].

The aim of this study was to compare the quality of minority patients’ transitions from hospital to community care with that of the non-minority Hebrew-speaking population. Specifically we tested the role of patient–provider language concordance in facilitating minority patients’ care transitions.

## Methods

### Setting

This prospective study was conducted at a large oncology center in a teaching hospital in the north of Israel with a catchment area encompassing two major minority groups: Arabic speakers (41% of the area’s population) and Russian speakers, namely immigrants (from 1990s onwards) from the FSU (14% of the area’s population) [29]. The Institutional Review Board of the Rambam Medical Center approved this study.

### Population

Patients were included in the study if they were scheduled to be discharged, were over the age of 18, spoke Hebrew, Arabic or Russian, were not receiving hospice care, and were cognitively able to answer a questionnaire. Recruiters were proficient in Hebrew, Arabic or Russian. Data were collected from January to March 2009. Of the 122 patients approached, 22 refused to participate due to fatigue or complaints of not feeling well enough to complete a questionnaire. Patients who refused participation were similar in their demographic characteristics to those who were included in the study (40% female, 30% above age 65). Of the 100 patients recruited 34 were Hebrew speakers, 34 Russian speakers and 32 Arabic speakers (according to native language, which was the preferred spoken language reported by respondents). Respondents were those discharged from hospitalization at one of the three oncology departments at the medical center. Eight respondents were lost to follow-up: three died before the two-week telephone follow-up and five declined to complete the phone survey. The final sample was 92 patients (31 Hebrew, 31 Russian, and 30 Arabic speakers). The study was originally powered to detect differences in ratings between Arabic and Russian speakers, with the application of G power analysis for two independent means [33] based on estimates of a >15% difference between groups [34], with power of 80% and alpha = 0.5

### Data collection

Upon patients’ consent, a questionnaire examining preparation for discharge from the hospital was administered. Also, structured observations were conducted during the patient discharge process. Two weeks after discharge patients were surveyed by phone regarding the transition from hospital to the community and their primary care physician’s (PCP) post-discharge care. Data were collected in the patients’ native language (Hebrew, Arabic or Russian).

To assess provider–patient language concordance we asked the patients to state the language in which the pre-discharge explanations and/or the final discharge briefing were given (Arabic, Russian or Hebrew). Provider–patient language concordance was dichotomized as 1 = language concordance at the pre- and/or the final discharge briefing, or 0 = no language concordance at both the pre- and the final discharge briefings.

To control for factors that could affect patients’ understanding of the transition process, we constructed several measures that reflect the extent of the discharge preparation process: (1) a pre-discharge questionnaire (before the patient was handed the discharge letter) and (2) a structured observation of the explanations provided during the discharge briefing, when the patient was handed the discharge summary letter immediately before leaving the hospital. We used this multi-method approach to ensure that we captured explanations provided to patients before and during the actual final discharge briefing.

We summarized the above two indices to capture the extent of overall explanations provided during the pre-discharge briefing (e.g., “Did the staff provide information on post-discharge self-care?”) and the final discharge briefing (e.g., “Was the medication list reviewed?”). Each item addressed received 1 point. Scores of this *general discharge explanations index* ranged from 0–18 (Cronbach’s alpha 0.70).

In addition, we developed a *quality of the discharge briefing index* based on observations of the duration and location of the explanation, and whether the patient was given the opportunity to ask questions. Scores ranged from 0 to 5 (Cronbach’s alpha 0.54).

The post-discharge survey assessed patients’ perceptions of the care transition experience by the Care Transition Measure (CTM) [35], whether the patient visited the PCP, and the PCP’s explanation of discharge recommendations. We examined whether the PCP reviewed the discharge recommendations on medications, follow-up care and referrals. We constructed an index to summarize the extent of explanations provided by the PCP. Scores of the *PCP review of discharge recommendations index* ranged from 0 to 5 (Cronbach’s alpha 0.89). We assessed overall health status (The Physical Component Score (PCS) and the Mental Component Score (MCS)) using the SF-12 v.2 [36]. Self-reported demographic characteristics included age, gender, education level and economic status.

Our primary outcome was patients’ assessment of the care transition process using the CTM, a 15-item measure of the transitions from hospital to community care. Patients are asked to rate their understanding of how to manage their health, how far their preferences have been taken into account, and if they understand their medication treatment. Answers are rated on a 4-point scale from “strongly disagree” to “strongly agree”. The CTM scale score (0 to 100) has evinced high internal consistency and reliability (Cronbach’s alpha 0.93) [37]. The CTM had previously been translated into Hebrew, Arabic and Russian. The translated versions showed high reliability and validity (Cronbach’s alpha 0.90-0.94) [38].

### Statistical analysis

We conducted univariate analyses using chi-square tests for categorical variables, and t-tests for continuous variables, to determine differences in patient characteristics and the CTM scores among the patient groups, for the whole sample and for each subgroup (Hebrew, Russian or Arabic speakers), and to determine differences in CTM scores by the demographic variables among the three groups. T-tests served to determine differences in CTM scores by language concordance among the three patient groups (dichotomous). For the Hebrew speakers all discharges were conducted in Hebrew, so these respondents were classified as receiving “language-concordant” care. Bivariate analyses (Pearson correlations) between CTM scores and continuous independent variables were conducted among the three patient groups.

A multiple linear regression model was constructed to test the association between each independent variable and the CTM score. The variables entered into the models were those that were significantly associated with the CTM score in the univariate analysis: language concordance (0 = no; 1 = yes); native language (Hebrew, Russian or Arabic); PCP review of discharge recommendations index (continuous); general discharge explanations index (continuous); quality of the discharge briefing (continuous); and physical and mental health status (continuous). Variables were entered into the model using stepwise forward selection. Data analysis was by SPSS statistical software version 17.

## Results

Table 1 presents the characteristics of each of the three patient groups. The Arabic speakers were younger, had lower levels of education, reported poorer economic status and had poorer mental health status than the Hebrew and Russian speakers. The Russian speakers had significantly higher education levels than the Hebrew and Arabic speakers, and significantly lower physical health status. Overall, 30 (49%) of the minority patients’ discharges were determined to be language concordant: for 17 (55%) of the Russian speakers and for 13 (43%) of the Arabic speakers.

**Table 1:** Sample characteristics and language concordance, n(%)*

	**Total**	**Hebrew**	**Russian**	**Arabic**	P value^**^
	**92 (100%)**	**31 (33.7%)**	**31 (33.7%)**	**30 (32.6%)**	
**Age (Mean, SD)**	59.3 (15.9)	59 (14.8)	67.2 (10.9)	51.4 (17.8)	<0.05†, <0.001†
Gender (female)	43 (46.7)	12 (38.7)	20 (64.5)	11 (36.7)	<0.05‡
Education (High-School or less)	50 (54.3)	21. (67.7)	3 (9.7)	26 (86.7)	<0.001‡
Economic status					
Poor and Very Poor	33 (35.9)	11 (35.5)	8 (25.8)	14 (46.7)	>0.05
Like others	42 (45.7)	13 (41.9)	18 (58.1)	11 (36.7)	
Good and very good	17 (18.5)	7 (22.6)	5 (16.1)	5 (16.7)	
Health status (mean, SD)					
PCS	32.8 (10.8)	31.9 (11.3)	31.4 (11.1)	35.2 (10.2)	>0.05
MCS	34.4 (12.5)	33.4 (11.8)	38.2 (14.8)	31.3 (09.1)	<0.05¶
Language concordance (yes)	61 (66.3)	31 (100)	17 (54.8)	13 (43.3)	<0.001‡

SD: standard deviation; PCS: physical component score; MCS: mental component score (SF-12 v.2).

*% Varies due to missing responses.

**p-values derived from t-test for continuous variables and chi-square tests for categorical variables.

†Significant difference between Hebrew and Russian speakers (p < 0.05) and between Russian and Arabic Speakers (p < 0.001).

‡Significant difference among all three groups.

¶Significant difference between Russian and Arabic speakers.

CTM score of the Arabic speakers was higher (mean = 58.82; SD = 14.91) than that of the Russian speakers (mean = 54.94; SD = 15.93) and significantly higher than that of the Hebrew speakers (Mean = 51.18; SD = 10.86) (Table 2). Significant gaps were found in certain items (p < 0.05): scores for receiving an easily understood written plan were 1.53, 1.94 and 1.23 for the Arabic, Russian and Hebrew speakers respectively; scores for having confidence to perform self-care activities were 2.80, 1.77, and 2.42 for the Arabic, Russian, and Hebrew speakers respectively.

**Table 2:** Care Transition Measure score (CTM) according to demographic variables

		**CTM (Mean, SD)**		
	**Total**	**Hebrew**	**Russian**	**Arabic**
CTM score^*^	54.94 (14.25)	51.18 (10.86)	54.94 (15.93)	58.82 (14.91)
**Gender**				
Male	54.02 (14.85)	53.22 (11.51)	56.55 (14.99)	57.54 (15.6)
Female	55.60 (13.08)	47.07 (9.21)	54.05 (16.73)	61.01 (14.06)
	t = -0.53; p = 0.601	t = 1.60; p = 0.121	t = -0.41; p = 0.684	t = -0.61; p = 0.549
**Education**				
High-School or less	56.40 (13.95)	51.00 (11.14)	61.40 (6.89)	60.26 (15.32)
Above high school	53.12 (14.38)	51.56 (10.83)	54.24 (16.53)	49.44 (7.56)
	t = 1.11; p = 0.271	t = 0.13; p = 0.90	t = -0.74; p = 0.47	t = -1.37; p = 0.18
**Economic status**				
Poor and very poor	55.35 (11.67)	52.93 (8.54)	50.38 (12.67)	60.32 (12.20)
Like others	52.43 (13.75)	50.09 (10.21)	51.62 (14.16)	56.36 (17.28)
Good and very good	60.13 (18.4)	50.48 (15.85)	74.18 (14.66)	60 (18.86)
	F = 1.85; p = 0.164	F = 0.21; p = 0.811	F = 5.75; **p = 0.008**	F = 0.22; p = 0.802
**Age**	r = -0.13; p = 0.223	r = -0.07; p = 0.705	r = -0.11; p = 0.575	r = -0.10; p = 0.616
**PCS**	r = 0.22; **p = 0.032**	r = 0.25; p = 0.184	r = 0.34; p = 0.060	r = 0.04; p = 0.847
**MCS**	r = 0.14; p = 0.195	r = 0.45; **p = 0.012**	r = 0.01; p = 0.975	r = -0.18; p = 0.356

SD = standard deviation; t = t-test for independent samples; F = one way ANOVA; r = Pearson correlation.

*T-test for differences between groups: Hebrew-Russian p = 0.283; Hebrew – Arabic p = 0.026; Arabic-Russian p = 0.330.

Significant values are bolded.

The results in Table 2 indicate that the demographic variables age, gender, economic status and education were not statistically significantly associated with the CTM score in each of the three groups, except economic status in the Russian speakers (F = 5.75; p =0.008). The Physical Component Score (PCS) of the SF-12 was moderately correlated with the CTM score for the entire sample (r = 0.22; p = 0.032), although it did not reach statistical significance in each group separately. The CTM score was significantly correlated with the Mental Component Score (MCS) for the Hebrew speakers only (r = 0.45; p =0.012).

Table 3 presents the CTM scores by language concordance. Scores of patients who did and did not receive language-concordant care at discharge showed a significant statistical difference (p < 0.05). While Arabic speakers who received language-concordant care during discharge had the highest CTM scores (mean = 69.09; SD = 12.46), patients without language-concordant care had low CTM scores (mean = 49.83; SD = 14.9). Additionally, language concordance was associated with high CTM scores among Russian speakers, although overall this group had the lowest CTM scores (60.03 in language-concordant versus 48.80 in non-language-concordant discharges, p < 0.05).

**Table 3:** Care Transition Measure score (CTM) according to the independent variables

		**CTM (Mean, SD)**		
	**Total**	**Hebrew**	**Russian**	**Arabic**
Language concordance (Total)^*^				
Yes	57.53 (13.28)	**-**	60.03 (9.24)	69.40 (14.41)
No	49.83 (14.93)	**-**	48.80 (20.13)	50.72 (9.30)
	t = 2.52; **p = 0.014**	**-**	t = 2.07; **p = 0.048**	t = 4.31; **p = 0.001**
**Quality of discharge briefing index (range: 0-5)**	3.07 (0.96)	3.35 (1.082)	2.94 (0.892)	2.90 (0.845)
	r = 0.54; **p = 0.001**	r = 0.52; **p = 0.003**	r = 0.68; **p = 0.001**	r = 0.70; **p = 0.001**
**General discharge explanations index (range: 0-18)**	8.38 (3.06)	8.52 (3.15)	8.52 (3.41)	8.10 (2.62)
	r = 0.26; **p = 0.013**	r = 0.46; **p = 0.009**	r = 0.26; p = 0.158	r = 0.16; p = 0.41
**PCP review of discharge recommendations index (range:0-5)**	2.77 (2.07)	2.32 (2.18)	3.32 (1.92)	2.67 (2.04)
	r = 0.28; **p = 0.007**	r = -0.10; p = 0.62	r = 0.47; **p = 0.007**	r = 0.39; **p = 0.036**

SD = standard deviation; t = t-test for independent samples; r = Pearson correlation.

*Total exceeded the sum number in the Russian and Arabic speaking groups as Hebrew speakers (N = 31) were categorized as have received language concordant care.

Significant values are bolded.

The average score of the general discharge explanation index (the measure of discharge explanations at the pre-discharge and the final discharge briefings) was 8.38 (SD: 3.03, range 0–18). The majority of discharges included explanations about *taking medication*s (67 patients: 73%) and provided *instructions for follow-up care* (81: 88%). The aspects least frequently addressed were explanations of *medication side effects* (26: 28%) and *responsibility for follow-up treatment* (26: 28%). Eighty-one patients (88%) indicated that they delivered the discharge letter to the PCP. According to patient reports, PCP briefings most frequently addressed *providing explanations on medications* (52: 64%) and *explaining follow-up care* (47: 51%). Our specific examination of differences in the duration of explanations provided during the discharge briefing yielded no statistically significant differences among groups (p = 0.065).

In all three patient groups the quality of the discharge briefing was associated with CTM scores: extent of discharge explanations provided during discharge (r = 0.259; p = 0.013), post-discharge explanations by the primary care physician (r = 0.279; *P* = 0.007), quality of discharge briefings (r = 0.542; p < 0.001) and the physical component score (PCS) (r = 0.224; p = 0.032) (Table 3).

Table 4 shows the results of the multiple linear regression analysis for patient ratings of the care transition process. Age, gender, economic status and education were not significantly associated with the CTM score in the univariate analysis so they were omitted from the model. The covariates that were retained in the model (*P* < 0.05) were language concordance (β: 0.49; SE: 2.94), patients’ native language (Arabic vs. Hebrew – β: 0.471; SE: 3.01), (Russian vs. Hebrew – β: 0.307; SE: 2.91), quality of discharge briefing (β: 0.454; SE: 1.32), general discharge explanations index (β: 0.166; SE: 0.36), PCP review of discharge recommendations index (β: 0.158; SE: 0.54) and physical health status (β: 0.197; SE: 0.10). Mental health status measure was not retained in the model.

**Table 4:** Correlates of quality transitional care as measured by the CTM-15 (multiple linear regressions)

	**B**	**S.E**	**β**
**Language concordance between patient and physician/nurse**	7.48	2.94	0.24*
**Native language (Ref: Hebrew)**			
Arabic	14.25	3.01	0.47***
Russian	9.21	2.91	0.30**
**Quality of discharge briefing index**	6.75	1.32	0.45***
**General discharge explanations index**	0.77	0.36	0.16*
**PCP review of discharge recommendations index**	1.09	0.54	0.16*
**SF12v2: PCS**	0.26	0.10	0.20*

Variables entered into the stepwise regression model but not retained: Mental health status.

PCP: Primary Care Physician.

*p < 0.05; **p < 0. 01; ***p < 0. 001.

## Discussion

Our findings show for the first time, that the provision of language-concordant discharge explanations is associated with the quality of the transition process. Discharges in which the explanations were provided in the patients’ native language were rated significantly higher on the quality of care transitions questionnaire, controlling for the measure of explanations provided during discharge and at follow-up community care.

Not surprisingly – and as other studies show, minority patients understand care instructions best when they and physicians speak the same language [39-41]. The multicultural makeup of Israel’s population and its health care workforce [42] provides ample opportunities for the provision of language-concordant care. Nevertheless, as reported in this study language concordance is not always available. In about half of the discharges of minority patients (Arabic and Russian speakers) care was language-discordant. This deficiency resulted in significant failures by patients to understand their post-discharge care instructions, as evinced by the low CTM scores. As expected, we found some differences in the quality of the transition process among the various patient groups, including higher ratings of the quality of the discharge briefing by Hebrew speakers than by Russian and Arabic speakers.

In addition to language concordance, aspects significantly associated with assessment of the care transition process proved to be the measure of discharge explanation given during the discharge briefings and by the PCP at the post-discharge visit. These findings are consistent with previous studies on the importance of discharge planning and the post-discharge follow-up visit for the quality of the transition process [43].

Overall, CTM scores of Arabic and Russian speakers were higher than scores of Hebrew speakers. This finding possibly reflects expectations and/or cultural norms. Gross et al. [44] similarly found that Arabic and Russian speakers are likely to assess their care more favorably than native Hebrew speakers.

Importantly, language-discordant care for Russian speakers resulted in slightly lower CTM scores than for Arabic speakers or native Hebrew speakers. As CTM ratings are influenced by the explanations patients receive at the post-discharge PCP visit, a possible reason for these differences may be the way these explanations are given: for Hebrew and Arabic speakers they are likely to be in their native language. For Russian speakers language-concordant primary care is not always available. This possibility, however, deserves future research.

Previous research reported somewhat higher CTM scores for a culturally diverse nationally representative sample from the United States (~70%) [45] and for a similar population of Israeli oncology patients [34]. The difference in scores could be attributable to the difference in the timing of the assessment: in our study it was two weeks post- discharge, while in the aforementioned studies [34,45] it was up to 12 months post-discharge. Further research should assess the potential lag time bias in the assessment of the transition process.

Our findings have potentially important implications for post-discharge outcomes. A study on the influence of ethnicity and language concordance on physician–patient agreement showed that language is a considerable barrier to it [46]. Previous research [37] also showed CTM to be linked to re-hospitalizations and emergency room visits, and as is known, readmission reduction is one of the few quality measures which, if implemented properly, can serve as a catalyst for system integration [6]. As most Israeli hospitals, and many other health care systems worldwide [47], do not have adequate interpreter services, our results suggest that the quality of care transitions may be seriously jeopardized. Reliance on ad hoc solutions – for example, the chance existence of patient–provider language concordance – may lead to significant deficiencies in post-discharge care; instead, ensuring that language-concordant discharge briefings are provided should be formalized in policy and practice.

This study has several limitations. Although it was based on a small convenience sample of patients treated at a single, albeit large, oncology center, the demographic characteristics of the patient subgroups in the study were similar to those of the entire population of each subgroup [28]. Our findings identify the need to assess language barriers during care transitions in diverse settings.

Additionally, we did not assess health literacy directly. Patients who are non-native Hebrew speakers could still have excellent understanding of the Hebrew language and of medical instructions, while native Hebrew speakers may present low health literacy levels. Nonetheless, our findings show that language concordance is significantly associated with patients’ understanding of their care transition process – a finding of importance for clinical practice in which formal assessments of health literacy (beyond asking patients what their native language is) is not easily performed.

Further methodological limitations include the relatively low reliability of the *quality of the discharge briefing index*, which could be attributed to the small number of items (five). This measure should be further developed to more accurately describe the quality of the briefing during discharge. Nor did we assess patient–provider language concordance during the PCP visit. While such concordance may play a significant role in the quality of the post-discharge visit, it can be reasonably assumed that Hebrew speakers as well as Arabic speakers received care from PCPs who spoke their native language (as noted earlier, in Israel, Arabic speakers predominantly receive their primary care from Arabic speaking physicians). Still, the potential effect of such concordance should be evaluated in future studies.

## Conclusions

Patient–provider language concordance during the hospitalization discharge process and post-discharge PCP follow-up have important implications for the quality of care transitions. When providers speak the same language as their patients, they can tailor explanations to overcome barriers to patients’ understanding of their follow-up care. Mechanisms to assist patients from culturally diverse groups should be incorporated into care transitions, especially for patients receiving language-discordant care. Such mechanisms, in the form of mandated interpreter services and formal programs for improving cultural competency across the Israeli health care system, have recently been enacted by the Israeli Ministry of Health and should be evaluated in future studies. Future research should also assess these findings in other culturally diverse groups and in relation to post-discharge outcomes.

## Competing interests

The authors declare that they have no financial or personal relationship(s) which may have inappropriately influenced them in writing this paper.

## Authors’ contributions

NR conceived the study and carried out all phases of data collection, data analysis and drafting of the manuscript. HA advised on study procedures and coordination, and approved the final manuscript. ES participated in study design, advised on data analysis, and helped draft and revise the manuscript. All authors read and approved the final manuscript.

## Authors’ information

NR conducted this study as part of her MA thesis in management of health administration (MHA) at the School of Public Health, Faculty of Social Welfare and Health Sciences at the University of Haifa. HA, PhD, is Director of Nursing at the Rambam Medical Center. ES, PhD, is a Senior Lecturer at the Cheryl Spencer Department of Nursing, Faculty of Social Welfare and Health Sciences, University of Haifa, and was the thesis advisor for this work. ES also serves as a senior consultant to the Health Policy Planning department at the Chief Physician’s office of Clalit Health Services and is the Co-editor-in-Chief of the *International Journal for Equity in Health*.
